# Factors associated with no apparent coronary artery disease in patients with type 2 diabetes mellitus for more than 10 years of duration: a case control study

**DOI:** 10.1186/s12933-015-0307-z

**Published:** 2015-10-31

**Authors:** Mukund P. Srinivasan, Padmanabh K. Kamath, Narayan M. Bhat, Narasimha D. Pai, Poornima A. Manjrekar, Chakrapani Mahabala

**Affiliations:** Department of Internal Medicine, Kasturba Medical College, Manipal University, Mangalore, Karnataka 575001 India; Department of Cardiology, Kasturba Medical College, Manipal University, Mangalore, Karnataka India; Department of Biochemistry, Kasturba Medical College, Manipal University, Mangalore, Karnataka India

**Keywords:** Coronary artery disease, Insulin resistance, No apparent coronary artery disease, Type 2 diabetes mellitus

## Abstract

**Background:**

Type 2 diabetes mellitus is an important risk factor in the development of coronary artery disease (CAD) and is often associated with severe disease. However, this risk is not uniform, some patients remain free of CAD even after many years of treatment for diabetes. The present study was aimed to identify the factors that are associated with a favorable CAD profile.

**Methods:**

A case–control study of 76 patients with type 2 diabetes mellitus who were on treatment for more than 10 years duration and undergoing a coronary angiogram for the evaluation of clinically suspected CAD at a tertiary care hospital were recruited for the study. The presence and absence of significant CAD was determined after a coronary angiogram. Clinical history, and anthropometric and biochemical parameters were analyzed. Insulin resistance was determined by the Homeostasis Model Assessment. Multiple logistic regressions were done to find out the factors associated for a favorable CAD profile.

**Results:**

The difference in HOMA-IR (2.37 ± 0.69 VS 3.77 ± 1.64, p < 0.001) and urine microalbumin (24.15 ± 32.16 VS 82.72 ± 117.70, p = 0.004) were found to be statistically significant among those who did not have CAD when compared to those who had CAD. The difference in lipid profile, HbA1C, fasting blood sugar, BMI, waist hip ratio, waist and hip circumference was not significant. The adjusted odds ratio for insulin resistance less than 2.5 (OR 9.09, 95 % CI 1.91–41.83, p = 0.005), females (OR 7.91, 95 % CI 1.55–40.38, p = 0.013) and microalbumin <20 mg/l (OR 4.57, 95 % CI 1.17–17.85, p = 0.029) were independently associated with normal coronaries. The adjusted odds ratio for lipid profile, BMI, blood pressure and HbA1C were not significant.

**Conclusions:**

HOMA-IR less than 2.5, microalbuminuria less than 20 mg/l and females are the factors appear to be associated with no apparent CAD.

## Background

Type 2 diabetes mellitus is an independent risk factor for coronary artery disease and often associated with severe and extensive disease [1]. The phenomenon for the development of coronary artery disease (CAD) in type 2 diabetes mellitus is multifactorial and the known risk factors account for about 25 % of the disease [2]. Even with significant advances in anti-glycemic therapies, the risk of macrovascular complications is not reduced in type 2 diabetes mellitus [3].

In general, individuals with type 2 diabetes mellitus have to be treated aggressively for better cardiovascular outcome. However, diabetes itself being a heterogeneous disease, [4] it has been seen that development of cardiovascular complications is not uniform in all patients with diabetes mellitus. Most of the diabetic populations are prone to get CAD in spite of conventional risk factors being controlled and some patients remain free of CAD despite many years of treatment for diabetes.

Recent rethinking has led to the conclusion that, the treatment for type 2 diabetes mellitus should be individualized. A paradigm shift is observed in the management of type 2 diabetic patients towards individualized treatment. A study of risk factors associated with favorable CAD profile will help in identifying low risk patients and will also help to control these factors in future generation of CAD in diabetic patients. By identifying a specific parameter where individualized treatment can be performed light might be thrown on reducing the vascular complications in type 2 diabetes mellitus.

Thus the present study was designed to evaluate the role of insulin resistance along with other clinical markers in type 2 diabetic patients who were angiographically documented with and without significant CAD, and on treatment for diabetes for more than 10 years.

## Methods

A case control study of 76 consecutive patients with type 2 diabetes mellitus, who were on treatment for a duration of more than 10 and who underwent a coronary angiogram for the evaluation of clinically suspected CAD [Those presenting with atypical angina and Treadmill test (TMT)–positive] at a Kasturba Medical College hospital, Mangalore, India were recruited from June 2013 to September 2014, after obtaining the written informed consent. The patients were selected randomly from a sample of 243 patients who participated in an ongoing cross-sectional study [5].

The details of diabetic duration were obtained from patients through their past medical history or from pervious hospital records and from personal history of diabetes. The study was restricted to a population between the ages of 45 and 65 years, since studies show that beyond 65 years of age the extent and degree of CAD remains same in all the population [6].

From the cohort of 243 patients, 76 patients were found to have a diabetic duration of more than 10 years. 38 controls and 38 cases were selected by computer generated randomization. Individuals were divided into control and cases based on the presence and absence of a significant CAD which was documented after a coronary angiogram. The presence of significant CAD was defined as any visible stenosis greater than 50 % on angiography in at least one major coronary artery [7], and those with stenosis <20 % or luminal irregularities in any of the epicardial arteries were defined to have ‘No Apparent CAD’ [8]. Based on the angiographic findings, 38 patients were found to have CAD considered as cases and the remaining 38 did not have significant CAD and served as controls.

Type 2 diabetes mellitus was diagnosed on the basis of glycated hemoglobin (HbA1c) levels and fasting blood glucose, according to the American Diabetes Association criteria (all T2DM individuals had HbA1c levels >6.5 % and fasting blood glucose >126 mg/dl) [9].

Those patients satisfying the ADA criteria for type 2 diabetes mellitus, who were on treatment for a duration of more than 10 years and those who had undergone a coronary angiogram were included in the study. Patients with known cases of chronic kidney disease, valvular heart disease, thyroid disorders, less than 10 years of diabetic duration, those who were on exogenous insulin administration and on steroids were excluded from the study. The study was conducted after obtaining the ethical approval from the Institutional Ethics Committee.

All the clinical findings were noted. Anthropometric measurements such as height, weight, waist and hip circumference were noted as per standard norms [10]. Body Mass Index (BMI) and waist hip ratio were calculated. Biochemical parameters such as fasting blood sugar, fasting insulin, fasting lipid profile, and urine microalbumin were analyzed as previously described by Srinivasan et al. [5]. The glycated hemoglobin was analyzed by high performance liquid chromatography (HPLC). The degree of insulin resistance was measured by the Homeostasis Model Assessment (HOMA 2) computerized method [11]. In large epidemiological studies, the use of HOMA-IR has been shown to correlate well with gold standard hyper-insulinemia euglycemic glucose clamp technique for the measurement of insulin resistance [12]. Individuals were said to be insulin resistant if HOMA-IR value was 2.5 or above, which is considered to be the optimal cutoff value particularly with respect to the Indian population [13]. In order to achieve a steady state and to avoid changes in insulin resistance that may occur due to acute stress of the disease and due to angiographic procedure, the blood test was done 2 weeks after the performance of the coronary angiogram [14].

The study population was also followed up for 1 year looking for any Major Adverse Cardiovascular Events (MACE) at 1 year. MACE is defined as cardiac death, nonfatal myocardial infarction, or target lesion revascularization [15]. Accordingly, at the end of one-year the MACE rate was calculated.

### Statistical analysis

Data were presented as mean ± SD. The categorical variables were represented as proportions/percentages. The normality assumption for continuous variables was evaluated by the Kolmogorov–Smirnov test. The independent sample t test was carried out in order to compare the means between the cases and the controls. A multiple logistic regression analysis with the absence of significant CAD as a dependent variable was performed. The adjusted odds ratio for insulin resistance and other biochemical risk factors was estimated and the results were represented as adjusted odds ratio (OR) and 95 % confidence interval (95 % CI). P < 0.05 was considered to be statistically significant. Analysis was carried out using the Statistical Package for Social Sciences (SPSS Version 16, Chicago, IL, USA).

## Results

Seventy-six patients with type 2 diabetes mellitus who were on treatment for a duration of more than 10 years were analyzed in this case control study. The mean age of our study population was 56.61 ± 5.36. The presence of significant CAD was seen in 38 patients (50 %) and 38 patients did not have significant CAD (50 %). In this present study, the male population of 50 (66 %) was predominantly high compared to female population of 26 (44 %). Among the study population, 22 (58 %) males and 16 (42 %) females did not have significant CAD and 28 (74 %) males and 10 (26 %) females were found to have CAD respectively. About 15 (40 %) were hypertensive and 23 (60 %) did not have hypertension in those without CAD and 21 (55 %) were hypertensive and 17 (45 %) did not have hypertension in subjects presenting with CAD.

The clinical characteristics of diabetic patients with and without CAD are shown in Table 1. There was a significant difference in the mean value of HOMA-IR (p < 0.001), microalbumin (p = 0.005), fasting blood sugar (p = 0.007) and ejection fraction (p < 0.001) in patients with CAD when compared to patients without CAD (Table 1). There was no significant difference in the mean value of fasting lipid profile, waist circumference and body mass index between the two groups (Table 1).

**Table 1:** Clinical characteristics of type 2 diabetic patients with and without CAD

Variable	Type 2 diabetic patients with CAD (N = 38)	Type 2 diabetic patients without CAD (N = 38)	p value
Duration of diabetes (years)	17.02 ± 4.38	14.50 ± 3.76	0.009
Fasting blood sugar (mg/dl)	210.87 ± 61.77	170.84 ± 63.66	0.007
Fasting Insulin (IU/µl)	22.83 ± 7.95	15.95 ± 5.26	<0.001
HOMA-IR	3.77 ± 1.63	2.37 ± 0.69	<0.001
HbA1c	9.72 ± 2.08	9.23 ± 1.69	0.261
Total cholesterol (mg/dl)	169.15 ± 64.06	165.21 ± 42.49	0.753
TC/HDL	4.26 ± 1.56	4.28 ± 1.70	0.967
LDL-C (mg/dl)	106.78 ± 51.70	102.16 ± 40.73	0.667
Microalbumin (mg/l)	82.22 ± 117.70	24.15 ± 32.61	0.004
Body mass index (kg/m^2^)	23.09 ± 2.54	23.33 ± 3.03	0.714
Waist circumference (cm)	86.99 ± 8.56	87.07 ± 6.73	0.965
Ejection fraction	47.84 ± 7.94	59.15 ± 3.31	<0.001

*HOMA*–*IR* homeostasis model assessment–insulin resistance, *TC*/*HDL* total cholesterol/high density lipoprotein ratio, *LDL* low density lipoprotein, *HbA*_*1c*_ hemoglobin A1C

The multiple logistic regression analysis was performed with the presence/absence of significant CAD as the dependent variable and the following as predictive variables: HOMA-IR, hypertension, smoking, microalbuminuria, HbA1c, BMI, Low density lipoprotein- cholesterol (LDL), high density lipoprotein- cholesterol (HDL), total cholesterol to HDL ratio and waist circumference. HOMA-IR <2.5 (OR: 9.09, 95 % CI 1.91–41.83, p = 0.005), females (OR 7.91, 95 % CI 1.55–40.38, p = 0.013) and microalbumin <20 mg/l (OR: 4.57, 95 % CI: 1.17–17.85, p = 0.029) were independently associated with the absence of significant CAD. The adjusted odds ratio for lipid profile, BMI, hypertension and waist circumference were not significant (Table 2).

**Table 2:** Logistic regression analysis for predicting favorable CAD profile in type 2 diabetic patients who are on treatment for more than 10 years of duration

Variable	Wald	B	Adjusted odds ratio	p value	95 % CI
Insulin resistance <2.5	8.03	2.20	9.09	0.005	1.97–41.83
Females	6.18	2.06	7.91	0.013	1.55–40.38
Microalbumin <20 mg/l	4.79	1.52	4.57	0.029	1.17–17.85
Duration of diabetes (years)	2.05	0.11	1.12	0.152	0.95–1.31
Fasting blood glucose (mg/dl)	2.20	0.008	1.00	0.137	0.99–1.01
TC/HDL	0.001	−0.03	0.97	0.969	0.21–4.35
LDL-C (mg/dl)	0.54	0.50	1.65	0.461	0.43–6.36
Hypertension	1.07	0.66	1.94	0.300	0.55–6.87
Age	0.03	−0.011	0.98	0.856	0.87–1.11
Body mass index (kg/m^2^)	0.97	0.81	2.26	0.323	0.44–11.38
Waist circumference (cm)	0.25	0.42	1.52	0.616	0.29–7.99

*TC/HDL* total cholesterol/high density lipoprotein ratio, *LDL* low density lipoprotein

At the end of 1 year, 3 (7.9 %) out of 38 subjects with No Apparent CAD, and 15 (41 %) out of 38 subjects with CAD developed MACE (p < 0.001). Based on HOMA-IR levels, 18 (38 %) out 47 subjects with HOMA-IR >2.5 developed MACE. The MACE was not observed in any of subjects with HOMA-IR <2.5 (p = 0.001).

## Discussion

Despite their being controlled for conventional risk factors of CAD, the majority of diabetic patients have an early onset of coronary artery disease and the involved vessels show severe disease. Most of the long term complications in diabetics are vascular in nature. Type 2 diabetic individuals are not homogenous in nature, in spite of many years of treatment for type 2 diabetes mellitus some patients remain free of CAD. Thus in the present study we wanted to find out which of these factors are associated with No Apparent CAD in type 2 diabetic patients who are on treatment for a duration of more than 10 years. In this population of diabetic patients who are on treatment for more than 10 years of duration, HOMA-IR <2.5, microalbumin <20 mg/l and females were independently associated with No Apparent CAD.

Much data suggests that insulin resistance has a central role in atherosclerosis and is subsequently associated with an elevated risk of cardiovascular disease [16, 17]. Altered insulin signaling in endothelial cells has emerged as an important mechanism for the increased susceptibility to cardiovascular disease [17]. Under physiological conditions, there is a delicate and balanced release of relaxing factors and endothelium-derived contraction factors that are apparently imbalanced in diabetes. Endothelial dysfunction which develops due to this alteration contributes to progressive atherosclerosis along with the proinflammatory state induced by insulin resistance [17].

At physiological levels, insulin initiates its action by binding to its specific cell surface receptor present in liver and skeletal muscle. The interaction between insulin and its receptors triggers the cascade of events associated with multiple physiologic or pathologic processes. Upon binding with its ligand, a series of structural conformational changes are initiated, resulting in the activation of intracellular tyrosine kinases and subsequent intracellular signal transduction, ultimately leading to the regulation of various important physiologic functions in the human body.

The mechanism of action of insulin is biphasic, insulin at basal state promotes a mitogenic pathway and the pulsatile action of insulin promotes a metabolic pathway. At normal physiological levels of insulin, the metabolic functions are mediated through the phosphatidylinositol (PI) 3-kinase pathway, playing a crucial role in GLUT-4 translocation, an important mechanism of insulin action; on the contrary, the mitogenic action of insulin is mediated through mitogen-activated protein kinase (MAPK) pathway, which mediates the non-metabolic, pro-inflammatory and proliferative effects of insulin [18].

In the case of insulin resistance, the response of target cells towards insulin is considerably diminished for metabolic (i.e., glucose metabolism) action, but with preserved mitogenic and anabolic actions. An acute rise in basal insulin is stimulatory, but a steady increase in insulin levels attenuates the target cells for glucose metabolism, through various mechanisms, by exerting its effects at the level of the receptor itself and at various sites outside the receptor as well. The constant elevated level of basal insulin, irrespective of its origin, usually leads to generalized insulin resistance [19]. The metabolic action of insulin is impaired while the mitogenic action is still ongoing. In other words, the phosphatidylinositol (PI) 3-kinase pathway is inhibited and MAPK pathway is still intact leading to proliferative, fibrotic changes in vasculature which explains the severity of the disease and the long segment disease seen in diabetic patients.

Studies have been reported where long term cardiovascular complications could be predicted by the measure of insulin resistance [20, 21] and subsequent study has also shown that elevated insulin resistance is also associated with severity of CAD in type 2 diabetes mellitus [5]. In the present study, low level of HOMA-IR is independently associated with favorable CAD profile in long standing diabetic population.

Insulin resistance which is one of the components of metabolic syndrome has shown to be relatively constant in type 2 diabetes mellitus whereas all the other conventional risk factors changes over a period of time. Even the United Kingdom Prospective Diabetes study over 6 years of conventional treatment for type 2 diabetes has shown that insulin sensitivity, the reciprocal of insulin resistance being constant with 62, 60 and 62 % at 0, 1 and 6 years [22–24]. Thus in clinical practice it is important to look for insulin resistance and identify the individuals with high insulin resistance in the beginning itself as insulin resistance is known to predict long term complications.

Gender is also known to be one of the additional risk factor associated with cardiovascular disease. The mean HOMA-IR in type 2 diabetic females without CAD was 2.73 ± 0.68 and 2.11 ± 0.59 in type 2 diabetic males without CAD. Vonbank et al. has shown that insulin resistance in females is associated with metabolic syndrome but not with angiographically determined CAD [25]. Despite of having HOMA-IR value greater than 2.5, similarly in our study we found that type 2 diabetic females are known to be associated absence coronary atherosclerosis.

Microalbumin, a risk factor for cardiovascular disease is known to be associated with a coronary endothelial dysfunction. The studies have shown that the coronary endothelial dysfunction correlates with increasing levels of microalbumin in type 2 diabetic patients [26]. However, several cross sectional and longitudinal studies have shown that the insulin resistance is independently associated with development of microalbuminuria in type 2 diabetes mellitus [27, 28].

Since insulin resistance precedes much before than microalbuminuria in type 2 diabetes mellitus, it is possible that an elevated insulin resistance contributes to the development of microalbuminuria in type 2 diabetes mellitus. In our study we found that the mean microalbumin levels was 18.62 ± 11.28 in patients with HOMA-IR less than 2.5, and thus lower levels of microalbuminuria was found to be associated with No Apparent CAD in long standing diabetes.

The microvascular complications are normally seen in long standing diabetes. There is enough evidence to prove that intense glycemic control has resulted in microvascular benefit [29] in long standing diabetes, but the ACCORD trial has shown that intense glycemic control did not yield protection against CAD particularly in those with long standing diabetes [30]. In a cross-sectional study, severe vascular changes in coronary arteries were observed in more than 5 years of type 2 diabetes and an elevated insulin resistance accounted for this most of the cardiovascular burden in more than 5 years of type 2 diabetes mellitus [31]. Thus the macrovascular complications are time dependent should be addressed at a much earlier stage of type 2 diabetes mellitus.

A recent observation by recent Kim et al., where Intravascular Ultrasound (IVUS) a gold standard method to identify any vulnerable plaques in coronary arteries, was used to study the coronary artery remodeling [32]. The findings from the study reveals that, those with HOMA-IR >2.5 had positive coronary artery remodeling when compared to those HOMA-IR <2.5 [32]. Thus individuals with high HOMA-IR (HOMA-IR >2.5) are susceptible to develop future cardiovascular events. In this present study we observed that, subjects with HOMA-IR <2.5 did not develop any form of adverse cardiac events at the end of one year even, when compared to those with HOMA-IR >2.5 which is in lines with findings from Kim et al.

The unique evolution of insulin resistance in type 2 diabetes mellitus will help us in identifying the patients with high HOMA-IR in the beginning itself. Since low levels of HOMA-IR is associated with No Apparent CAD in long standing diabetes, the patients with high IR should be managed aggressively in the initial phase of diabetes. Maintaining a normal blood glucose level in type 2 diabetics by use of either oral medications or insulin will only fulfill the guideline requirements and will not achieve any long term benefits. It will not decrease insulin resistance or hyperinsulinemia that is present in these patients. The ultimate goal should be to target the patients with high HOMA-IR at the very beginning and encourage them to adopt radical life style modifications and thus disrupt the cascade of insulin resistance leading to CAD.

### Limitations

The limitation of the study is a small sample size and a single center study. Another limitation of the study is its study design. Ideally, prospective studies need to be carried out to determine the role of insulin resistance in type 2 diabetic patients without CAD. Long term follow-up with HOMA-IR measured at the onset of the disease and compared with angiographic findings after a few years along with other known risk factors might allow us to evaluate the role of each factor associated with a favorable CAD profile. The hyper-insulinemia euglycemic glucose clamp technique is said to be the gold standard for the measurement of insulin resistance/hyperinsulinemia but due its practical inconvenience, HOMA-IR index was used which has shown a good correlation with hyper-insulinemic euglycemic clamp test. Intravascular ultrasound (IVUS) is to be a gold standard method to identify vulnerable plaques, but in this present study the Coronary Angiogram was used to define No Apparent CAD, where a discrepancy in the results might be possible. However, it still remains unclear what morphological features will best predict plaque rupture and which diagnostic technologies would reliably predict the pathological and clinical courses of a vulnerable plaque.

## Conclusion

The results of our study indicates that HOMA-IR less than 2.5 microalbuminuria less than 20 mg/l and females appears to be the factors associated with a favorable CAD profile even with the absence of comparable lipid and glycemic parameters in type 2 diabetes mellitus in patients on treatment for a duration of more than 10 years.
